# Downregulation of PIK3CB Involved in Alzheimer's Disease via Apoptosis, Axon Guidance, and FoxO Signaling Pathway

**DOI:** 10.1155/2022/1260161

**Published:** 2022-01-20

**Authors:** Zhike Zhou, Jun Bai, Shanshan Zhong, Rongwei Zhang, Kexin Kang, Xiaoqian Zhang, Ying Xu, Chuansheng Zhao, Mei Zhao

**Affiliations:** ^1^Department of Geriatrics, The First Affiliated Hospital, China Medical University, Shenyang, 110001 Liaoning, China; ^2^Cancer Systems Biology Center, The China-Japan Union Hospital, Jilin University, Changchun, 130033 Jilin, China; ^3^Department of Neurology, The First Affiliated Hospital, China Medical University, Shenyang, 110001 Liaoning, China; ^4^Computational Systems Biology Lab, Department of Biochemistry and Molecular Biology and Institute of Bioinformatics, The University of Georgia, USA; ^5^Department of Cardiology, The Shengjing Affiliated Hospital, China Medical University, Shenyang, 110004 Liaoning, China

## Abstract

**Objective:**

To investigate the molecular function of phosphatidylinositol-4,5-bisphosphate 3-kinase catalytic subunit beta (PIK3CB) underlying Alzheimer's disease (AD).

**Methods:**

RNA sequencing data were used to filtrate differentially expressed genes (DEGs) in AD/nondementia control and PIK3CB-low/high groups. An unbiased coexpression network was established to evaluate module-trait relationships by using weight gene correlation network analysis (WGCNA). Global regulatory network was constructed to predict the protein-protein interaction. Further cross-talking pathways of PIK3CB were identified by functional enrichment analysis.

**Results:**

The mean expression of PIK3CB in AD patients was significantly lower than those in nondementia controls. We identified 2,385 DEGs from 16,790 background genes in AD/control and PIK3CB-low/high groups. Five coexpression modules were established using WGCNA, which participated in apoptosis, axon guidance, long-term potentiation (LTP), regulation of actin cytoskeleton, synaptic vesicle cycle, FoxO, mitogen-activated protein kinase (MAPK), and vascular endothelial growth factor (VEGF) signaling pathways. DEGs with strong relation to AD and low PIK3CB expression were extracted to construct a global regulatory network, in which cross-talking pathways of PIK3CB were identified, such as apoptosis, axon guidance, and FoxO signaling pathway. The occurrence of AD could be accurately predicted by low PIK3CB based on the area under the curve of 71.7%.

**Conclusions:**

These findings highlight downregulated PIK3CB as a potential causative factor of AD, possibly mediated via apoptosis, axon guidance, and FoxO signaling pathway.

## 1. Introduction

Alzheimer's disease (AD), referring to an irreversible neurodegenerative disorder, is manifested in cognitive decline, along with behavioral and psychiatric abnormalities of varying extent [1, 2]. The core hallmarks of AD comprise intracellular hyperphosphorylated tau and extracellular amyloid-beta (A*β*) plaques, which progressively deteriorate with loss of neurons and synaptic elements [3–7]. Pathologically, A*β* peptides are derived from the continuous cleavage of amyloid precursor protein (APP) by *β*- and *γ*-secretases, known as the amyloidogenic pathway related to neurodegeneration [8, 9]. This is in competition with the nonamyloidogenic process of *α*-secretase cleaving APP that prevents A*β* formation by releasing soluble amyloid precursor protein alpha (sAPP*α*) [10]. Imbalance of these two pathways leads to A*β* accumulation, which further elicits early synaptic alterations and ultimately synaptic loss, a process thought to be regulated by phosphoinositide 3 kinase (PI3K) [11, 12]. Multiple isoforms of PI3K are existed in higher eukaryotes, which can be categorized into three classes (I-III) according to sequence homology and substrate preference [13]. The main function of PI3K is to catalyze the phosphorylation of phosphatidylinositol on the 3-hydroxyl group of the inositol ring, successively processed by isoforms from class III, II to I [14, 15]. Such products of phosphorylated lipids, in turn, provide an anchor for the assembly of downstream proteins that trigger complex intracellular signaling cascades [14]. As an important signaling molecular, dysregulation of PI3K is ubiquitously observed in the development of many diseases, including AD [16]. Despite of no cure presently available for the disease, the exploration of causative factors or crucial regulators (e.g., PI3K) associated with AD may delay or even prevent the occurrence and progression of AD.

Traditionally, class I PI3K is the most concerned isoform of PI3K family, which consists of a regulatory subunit and a catalytic subunit that play pivotal roles in cell proliferation, differentiation, and survival (or apoptosis) [17–19]. In terms of the catalytic subunit of class I PI3K, phosphatidylinositol-4,5-bisphosphate 3-kinase catalytic subunit beta (PIK3CB) is highly expressed in neurons implicated in synapse formation and cell cycle regulation [20, 21]. Several lines of evidence have demonstrated that PI3K signaling pathways are linked to the pathophysiology of AD. For instance, TREM2 alleviates neuroinflammation and cognitive impairment through PI3K/AKT/FoxO3a signaling in AD mice; conversely, this neuroprotective effect of TREM2 can be eliminated with PI3K inhibitors [22]. Similar results are also replicated by sulforaphene administration, which attenuates neuroinflammatory response and hyperphosphorylation of tau via modulating the PI3K/Akt/GSK-3*β* pathway [23]. To the best of our knowledge, the pathogenic mechanism whereby dysregulation of PIK3CB mediates AD is not well understood. Toward this, we sought to conduct a comprehensive genomic analysis on basis of gene expression profile and functional annotations [24], which might shed light on the molecular role of PIK3CB in the pathogenesis of AD.

## 2. Materials and Methods

### 2.1. Data Resources

Microarray RNA sequencing (RNA-seq) data from middle temporal gyrus of 78 human samples (46 AD patients and 32 age-matched nondementia controls) were accessible through Gene Expression Omnibus (GEO) Series accession number GSE109887 [25, 26]. Illumina HumanHT-12 V4.0 expression beadchip was used to detect the expression of 16,790 annotated genes with 31,700 probes. To reduce prediction error on cross studies, the normalization of gene expression profiles was processed with the *normalizeBetweenArrays* function in R package [27].

### 2.2. Gene Set Enrichment Analysis (GSEA)

GSEA is a widely used computational method assessing whether enrichment of a predefined gene set is statistically significant between two biological states [28]. During the process of functional annotation, 1000 permutations were set up to screen biological processes (BP) of gene ontology (GO) terms utilizing *ClusterProfler, enrichplot* and *GSEABase* packages [29]. The visualization of GSEA data was accomplished by *ggplot2* package. A *pvaluecutoff* of 0.05 was determined as the screening threshold for significant enrichment.

### 2.3. Differential Expression Analysis

Taken the average expression value of PIK3CB to be the boundary, enrolled samples were dichotomized into PIK3CB-low and PIK3CB-high cohort. Using *lmFit* and *eBayes* functions, we identified the differences of gene expression in AD/control and PIK3CB-low/high groups, respectively [30]. A fold change (FC) > 1.7 combined with a false discovery rate (FDR)-adjusted *p* cutoff of < 0.05 was adopted to define differentially expressed genes (DEGs) [31–33]. Analysis of two-dimensional hierarchical cluster was carried out by R software of *limma* package [32]. Volcano plot and heat map were employed to visualize the expression of DEGs in the screening and cluster analyses.

### 2.4. Coexpression Network Analysis

Following the default parameters of weight gene correlation network analysis (WGCNA), overlapping DEGs between AD/control and PIK3CB-low/high cohorts were disposed to create an unbiased coexpression network. The preponderance of WGCNA is that it converts intricate microarray data into gene coexpressed modules, providing insight into a signal network that may be associated with phenotypic traits of interest [34]. To ensure the reliability of network outcomes, sample clustering diagram was plotted to eliminate outliers with low inter-array correlation using *hclust* function. An appropriate soft-thresholding power of 12 was selected with *pickSoftThreshold* function to achieve the integral connectivity of gene coexpression modules, so that the network utmost approaches the authentic biological state [35]. Module eigengene (ME) is the first major element for a module, known as the module representative that accounts for the maximum possible variability of all genes in a module [36]. Correlation coefficients of all genes with each ME were calculated to construct a hierarchical clustering tree, by which branches of >30 genes were reassembled into coexpression modules with unique color tags [37, 38]. Functional enrichment analyses were performed to filtrate Kyoto Encyclopedia of Genes and Genomes (KEGG) pathways using *clusterProfiler* package.

### 2.5. Global Regulatory Network and Cross-Talking Pathways of PIK3CB

Genetic phenotype and intramodular connectivity were measured by gene significance (GS) and module membership (MM), respectively, the relationships of which were plotted in a scatter diagram adopting *verboseScatterplot* function [39]. Based on an online database of Search Tool for the Retrieval of Interacting Genes (STRING, http://www.stringdb.org/), the interaction between DEGs was explored by establishment of protein-protein interaction (PPI) network [40]. The *cytoscape* software was used to visualize the global regulatory network, as well as the cross-talking pathways of PIK3CB that were enriched by pathway enrichment analyses [41, 42].

### 2.6. Analysis of Receiver Operating Characteristic Curve (ROC)

ROC analysis was conducted to estimate the classifier performance of sequential output, including sensitivity and specificity parameters, as measured by the area under the curve (AUC) [43, 44]. Diagnostic performance of PIK3CB to distinguish AD cases from nondementia controls was assessed using *pROC* package in R. An AUC value of 100% indicated complete prediction, while 50% represented random selection.

### 2.7. Statistical Analysis

Continuous variables between AD patients and nondementia controls were compared by applying *t* test or the nonparametric Mann–Whitney *U* test. During the process of WGCNA analysis, the statistical relationship between gene coexpressed modules and phenotypic traits of interest was estimated by Pearson correlation coefficient (PCC) analysis. Using the approach of Hanley-McNeil test, predictive accuracy of PIK3CB was verified by ROC analysis, the result of which was quantified by AUC value. Two-tailed *p* values of < 0.05 were defined as the standard of statistical significance. All statistical analyses were conducted by employing GraphPad Prism software (version 8.3.1) and R package (version 3.6.2).

## 3. Results

### 3.1. Overall Research Design

**Figure 1:**
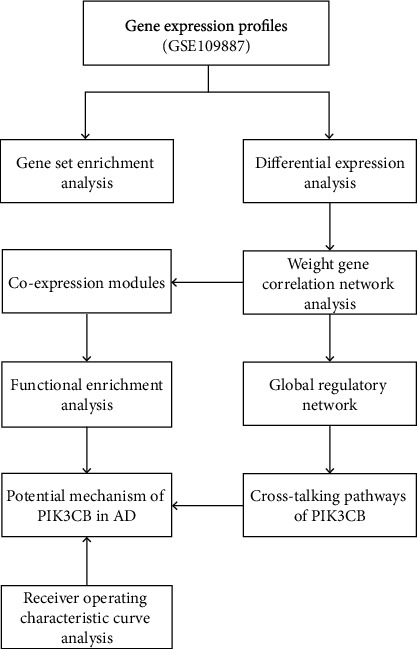
Flow chart of research design. AD: Alzheimer's disease.

The workflow diagram of this research was detailed in Figure 1. Herein, the GSE109887 dataset in GEO database was selected for bioinformatic mining. Subsequently, an integrative method of differentially expression analysis and clinical phenotype-based WGCNA was employed to build AD-related gene coexpression modules by a comparison between AD and nondementia controls. The cellular processes of each module were further enriched by functional enrichment analysis, which provided an understanding of the biological functions of coexpressed genes at the cellular level. Thenceforth, we constructed a global regulatory network based on module genes strongly interacting with AD and PIK3CB, wherein the cross-talking pathways of PIK3CB were identified. Additionally, diagnostic performance of PIK3CB in AD prediction was validated by ROC analysis.

### 3.2. Identification of Differentially Expressed Genes

**Figure 2:**
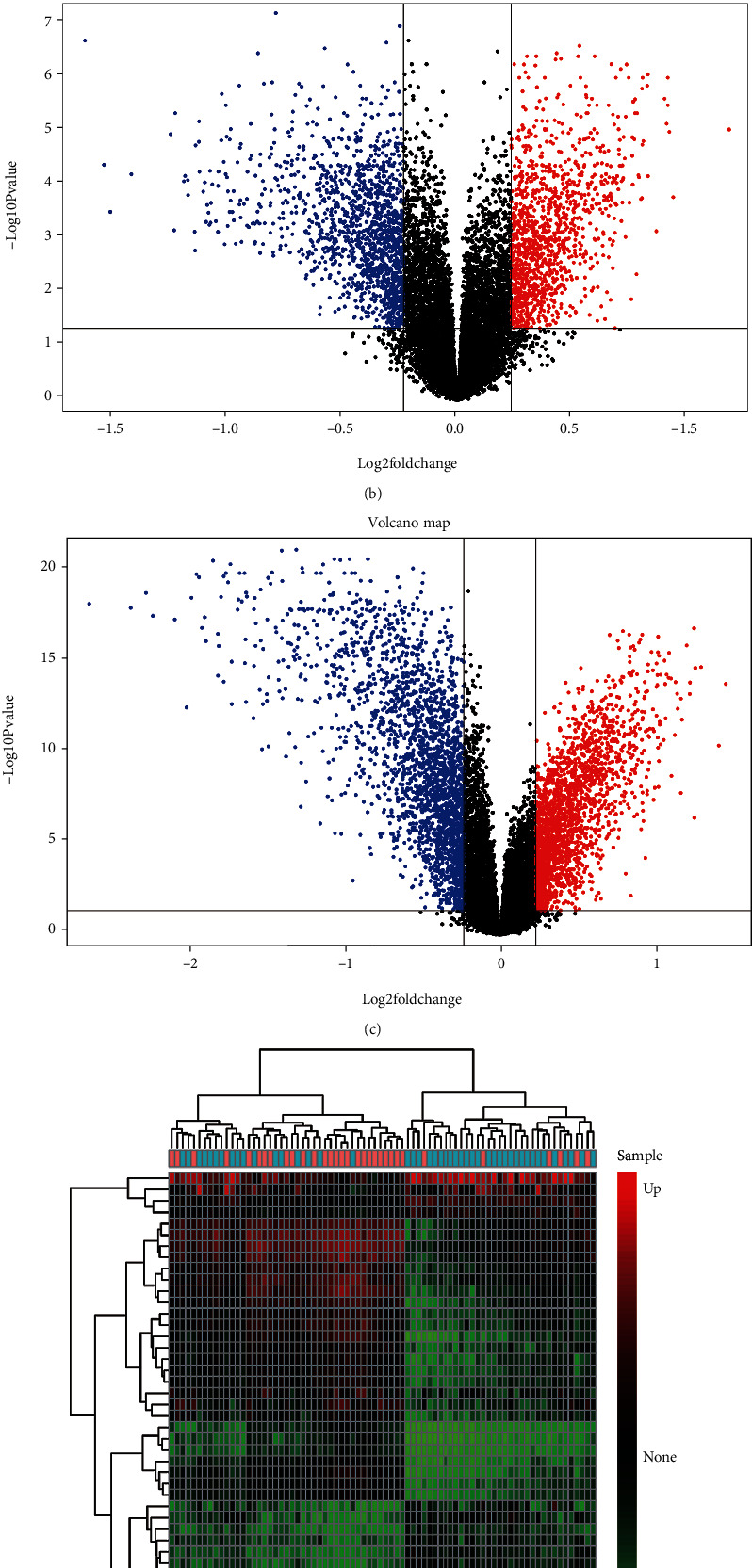
Differential expression analysis. (a) PIK3CB expression between AD and nondementia controls. ((b) and (c)) Volcano plots of DEGs in AD/control and PIK3CB-low/high cohorts: red indicates upregulated, while blue represents downregulated. (d) Heatmap of the first 25 up- and downregulated DEGs: red to green indicates gene expression alterations from upregulated to downregulated. AD: Alzheimer's disease; DEGs: differentially expressed genes.

Compared with controls, the mean gene expression of PIK3CB was significantly downregulated in AD patients (7.38 ± 0.44 versus 7.76 ± 0.53; *p* < 0.01) (Figure 2(a)). Through preliminary processing of microarray data, 16,790 background genes were generated for further DEG identification. Significant differences in the expression of 2,675 genes (1,616 up- and 1,850 downregulated) were screened in AD relative to nondementia cohort (Figure 2(b)), while 4,393 genes (2,096 up- and 2,297 downregulated) were differentially expressed in PIK3CB-low versus high group (Figure 2(c)). Subsequently, a total of 2,385 overlapping DEGs (1,093 up- and 1,292 downregulated) were filtrated between AD/control and PIK3CB-low/high groups. The expression of the first 25 up- and downregulated DEGs in AD versus nondementia controls was shown in heat map (Figure 2(d)), which exhibited substantially different biology between these two groups.

### 3.3. Coexpression Modules and Functional Enrichment Analysis

**Figure 3:**
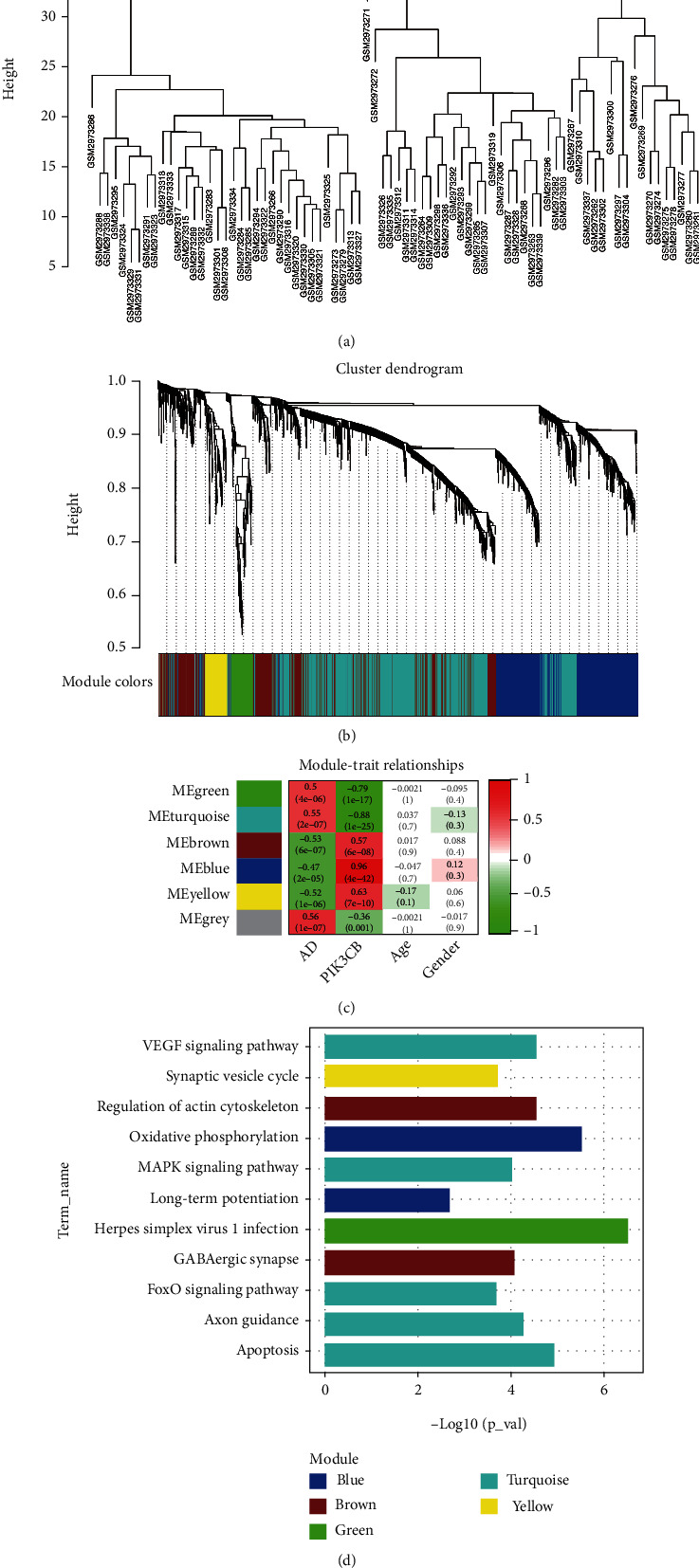
Weighted correlation network analysis. (a) Sample clustering to detect outlines. (b) Dendrogram of genes clustered by different module colors: gray represents noncoexpression genes. (c) Module-trait relationships: red to green indicates a positive to negative correlation of module eigengenes with phenotypes. (d) KEGG enrichment results of coexpression modules. AD: Alzheimer's disease; KEGG: Kyoto Encyclopedia of Genes and Genomes.

**Figure 4:**
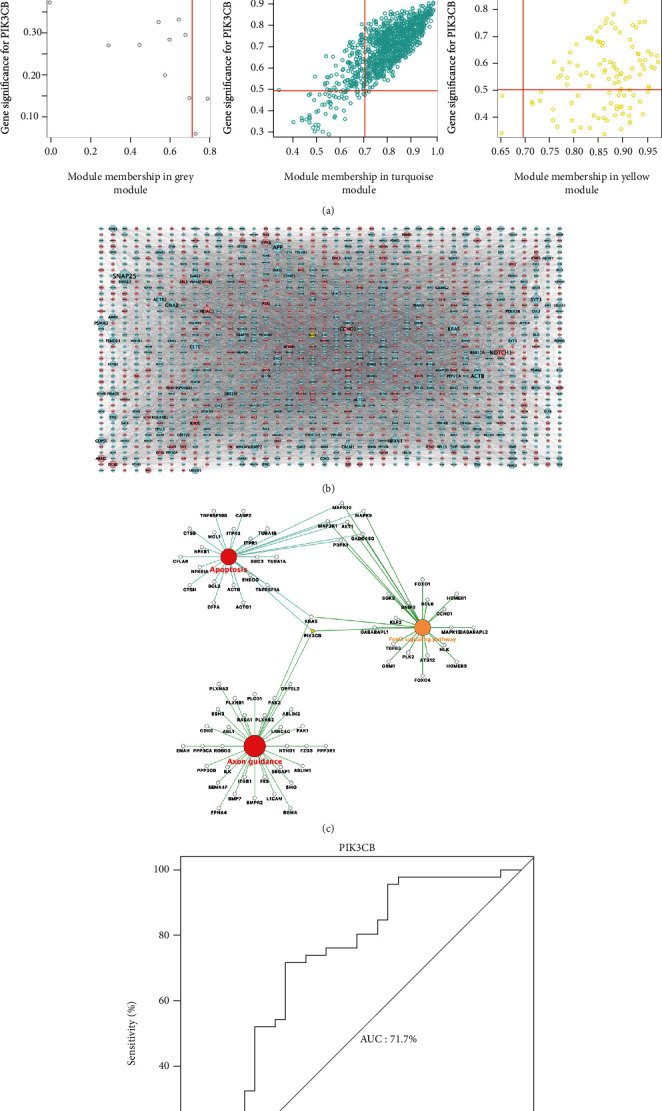
Module-pathway regulatory network and ROC analysis. (a) Scatter plot of module membership related to gene significance. (b) Global regulatory network based on PIK3CB-interacting genes: node size reflects the connectivity between genes; red indicates upregulated; blue and yellow represent downregulated. (c) Cross-talking pathways of PIK3CB: yellow is the downregulated PIK3CB. (d) AUC value of PIK3CB in predicting AD onset. AD: Alzheimer's disease; AUC: area under the curve; ROC: receiver operating characteristic curve.

During the process of hierarchical clustering detection of outliers by using average linkage, all samples passed the cut-off line (height = 45) and could be enrolled in subsequent gene coexpression network analysis (Figure 3(a)). As shown in Figure 3(b), coexpressed genes were clustered into five coexpression modules by WGGNA, implying that genes in each cluster were transcriptionally correlated; whereas the remaining noncoexpressed genes were grouped into a nonfunctional module, namely, the gray module. Heatmap of module-trait relationships (Figure 3(c)) revealed that green and turquoise modules had the significantly positive correlation with AD (green: correlation coefficient = 0.5, *p* = 4*e* − 06; turquoise: correlation coefficient = 0.55, *p* = 2*e* − 07) and negative correlation with PIK3CB expression (green: correlation coefficient = −0.79, *p* = 1*e* − 17; turquoise: correlation coefficient = −0.88, *p* = 1*e* − 25); while brown, blue, and yellow modules were negatively correlated with AD (brown: correlation coefficient = −0.53, *p* = 6*e* − 07; blue: correlation coefficient = −0.47, *p* = 2*e* − 05; yellow: correlation coefficient = −0.52, *p* = 1*e* − 06) and positively relevant to PIK3CB expression (brown: correlation coefficient = 0.57, *p* = 6*e* − 08; blue: correlation coefficient = 0.96, *p* = 4*e* − 42; yellow: correlation coefficient = 0.63, *p* = 7*e* − 10). Functional enrichment analysis of KEGG pathways (Figure 4(d)) showed that DEGs in blue module were enriched in oxidative phosphorylation and long-term potentiation (LTP); DGEs of brown module were involved in GABAergic synapse and regulation of actin cytoskeleton; DEGs in green module participated in herpes simplex virus 1 infection; DEGs of turquoise module were enriched in apoptosis, axon guidance, FoxO, mitogen-activated protein kinase (MAPK), and vascular endothelial growth factor (VEGF) signaling pathways; DEGs in yellow module were involved in synaptic vesicle cycle. Accordingly, the specific mechanistic pathways of coexpressed genes in each module in AD were functionally screened out.

### 3.4. Module-Pathway Regulatory Network and ROC Analysis

As shown in Figure 4(a), scatter diagram between MM and GS exhibited a significant correlation between intramodular connectivity and genetic phenotypes in the coexpression modules (blue: correlation coefficient = 0.96, *p* < 1*e* − 200; brown: correlation coefficient = 0.68, *p* = 6.2*e* − 65; green: correlation coefficient = 0.46, *p* = 4.8*e* − 07; turquoise: correlation coefficient = 0.81, *p* < 1*e* − 200; yellow: correlation coefficient = 0.25, *p* = 0.0079), suggesting that the five coexpression modules were prominently associated with low expression of PIK3CB in AD patients. To accurately identify the mechanistic pathways of PIK3CB in AD onset, DEGs strongly interacting with PIK3CB (meeting the inclusion criteria of MM > 0.7 and GS > 0.5) were extracted to construct a global regulation network (Figure 4(b)) based on STRING database. Using pathway enrichment analyses, further cross-talking pathways of PIK3CB, such as apoptosis, axon guidance, and FoxO signaling pathway, were determined in the network (Figure 4(c)). The ROC analysis (AUC = 71.7%) showed that low PIK3CB performed a good performance in differentiating AD cases from nondementia controls (Figure 4(d)).

### 3.5. GESA Validation in Biological Processes

**Figure 5:**
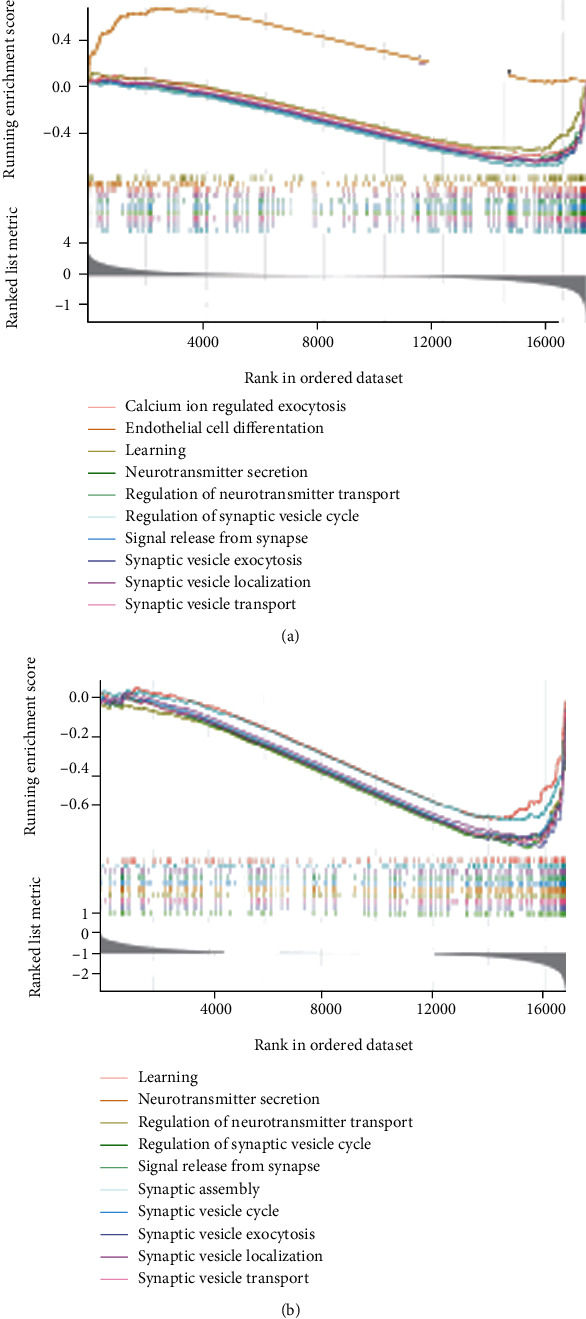
Gene set enrichment analysis. (a) Gene functional annotation of biological processes in AD. (b) Gene functional annotation of biological processes in PIK3CB-low group. AD: Alzheimer's disease.

To validate the potential functional mechanisms involved by PIK3CB in AD, GSEA was performed for BP enrichment according to a predefined gene set. The enriched BP in AD cohort (Figure 5(a)) was primarily linked to calcium ion regulated exocytosis, endothelial cell differentiation, learning, neurotransmitter secretion, signal release form synapse, and synaptic vesicle transport. The functions of core enrichment genes in the PIK3CB-low group (Figure 5(b)) were mainly distributed in BP of learning, synapse assembly, synaptic vesicle cycle, signal release from synapse, neurotransmitter secretion, and synaptic vesicle transport. These data underlined that low PIK3CB expression might contribute to the pathogenesis of AD through biological processes related to learning, neurotransmitter secretion, signal release form synapse, and synaptic vesicle transport.

## 4. Discussion

To improve our understanding of the AD phenotype modified by PIK3CB, we took advantage of the RNA-seq data to screen differentially expressed genes between AD and nondementia controls for integrative genomic analysis. The results of GSEA showed that functions of core enrichment genes were distributed in the BP of learning, neurotransmitter secretion, signal release form synapse, and synaptic vesicle transport. Historically, neurotransmitters have been described as the fundamental neurochemicals of signaling between presynaptic and postsynaptic neurons, responsible for the maintenance of synaptic and cognitive function [45–48]. Of particular note was that these biological processes were significantly associated with AD and low PIK3CB expression, indicating an important role of PIK3CB in AD pathogenesis. Consequently, this promoted us to construct a global regulatory network and coexpression modules of DEGs interacting with PIK3CB to illuminate the genomic mechanism of PIK3CB in the development of AD.

The findings emerging from WGCNA revealed that DEGs of coexpression modules were significantly correlated with AD and PIK3CB expression, which were enriched in apoptosis, axon guidance, LTP, synaptic vesicle cycle, FoxO, MAPK, and VEGF signaling pathways. There is evidence that APP aggregates in axonal growth cones, acting as a coreceptor for axon guidance and cell migration cues through its interaction with the extracellular matrix [49–51]. More specifically, sAPP*α*—the secreted product of *α*-secretase APP cleavage—antagonizes an inhibitor of axon guidance cue termed Sema3A, contributing to cell movement and axon outgrowth [52]. As supported by evidence in neural stem cell-derived neurons, inhibition of sAPP*α* secretion abolishes depolarization-induced neurite outgrowth and elongation [53]. Furthermore, sAPP*β*—the secreted product of *β*-secretase APP cleavage—not only promotes rapid neural differentiation but also loosens intercellular adhesion, which is thought to be critical for axonal outgrowth [54–56]. In addition, A*β*—the secreted product of *β*- and *γ*-secretase APP cleavage—is the major culprit of AD that impedes axon outgrowth by inducing allosteric collapse of growth cone, giving rise to impaired cognitive recovery [57, 58]. Altogether, the evidence presented above strongly points to a linkage between axon guidance and AD neurodegeneration. Axon guidance molecules including nerve growth factor (NGF) and insulin-like growth factor (IGF-1) have been shown to control axonal growth, a process that relies on tight regulation and localized activation of PI3K [59]. It has long been recognized that PI3K can both stabilize polymerized microtubules and interfere with microtubule polymerization, thus, modulating microtubule dynamics prerequisite for NGF-induced axon elongation [60]. Coincident outcomes have also been reported in an *in vitro* model of a biocompatible guidance device where a linear propagation of IGF-1 gradients sequentially directs axon outgrowth [61]. Notably, an essential step in the initiation of axonal outgrowth is precisely the activation of PI3K by IGF-1 and its receptors [62, 63]. By contrary, inhibition of PI3K enhances the response to growth cone collapse, which in turn hinders axon elongation [64, 65], consistent with our findings of low PIK3CB-mediated AD pathogenesis involving axon guidance.

An extensive array of studies support that apoptosis underlies the pathogenic mechanisms of neuronal cell death in AD. Analytic results of postmortem brain tissues provided evidence for a 50-fold increase of apoptosis in AD patients relative to nondementia controls [66]. As the most prevalent genetic risk factor for AD, apolipoprotein E4 (apoE4) has been identified to trigger apoptosis, leading to a series of detrimental consequences such as impaired neuroplasticity and cognitive decline [67]. This is confirmed by *in vitro* cell cultures that apoE4 induces an increase of apoptotic cell death in a subtype-specific manner [8, 68, 69]. Additionally, Takuma et al. found that mitochondrial dysfunction and endoplasmic reticulum- (ER-) induced stress had implications in the execution of apoptosis relevant to AD [70]. One plausible interpretation is that A*β* depletes ER Ca^2+^ reserves to promote excessive uptake of Ca^2+^ into mitochondria, causing cytosolic Ca^2+^ overload and thus to activate the mitochondrial-mediated apoptosis [71]. On the other hand, a growing body of research has directly or indirectly linked PIK3CB to apoptosis. In the rat model of subarachnoid hemorrhage, ErbB4-induced activation of PIK3CB increased yes-associated protein (YAP) expression, a terminal effector of Hippo signaling that dramatically improved neurological deficits and apoptosis; meanwhile, inhibition of ErbB4 or YAP knockdown could eliminate this anti-apoptotic effect [72]. Likewise, subsequent *in vitro* experiment on human glioma cell lines demonstrated that inhibition of PIK3CB by AZD6482 induced apoptosis and cell cycle arrest, as detected using flow cytometry with propidium iodide staining [18]. In fact, PIK3CB has attracted considerable attention as a selective survival factor for cancer therapy based on its critical role in apoptosis [73–76].

Taking into consideration that A*β* increases the production of reactive oxygen species (ROS), AD may be at higher risk due to mitochondrial oxidative stress through FoxO signaling pathway [77, 78]. Upregulation of FoxO transcriptional activity has been shown to drive the expression of genes related to antioxidative response by inducing epigenetic modification, contributing to intracellular metabolic homeostasis and oxidative stress clearance [79]. This activation, however, is supposed to be moderate rather than sustained, since the latter mode yields apoptosis instead of resistance to oxidative stress [80–82]. During the progression of AD, inhibition of FoxO disrupts mitochondrial energy metabolism through excessive release of ROS, resulting in impaired synaptic transmission and neuronal apoptosis [83, 84]. As supported by an *in vitro* experiment from animal AD models, A*β*-induced Ros activates p66Shc, an adaptor protein that triggers phosphorylation (i.e., inactivation) of FoxO, which aggravates the accumulation of oxidative stress and thus to neuronal and synaptic loss [85]. Relatedly, in response to oxidative stress stimuli, PI3K activates a downstream serine/threonine kinase, termed as protein kinase B (PKB) or Akt, which further negatively regulates FoxO transcriptional factors related to cell cycle and apoptosis [86, 87]. From our point of view, these data are in line with our computational results on the involvement of low PIK3CB in AD pathophysiology via apoptosis and FoxO signaling pathway.

Based on the scatter diagram of the relationship between MM and GS, DEGs strongly interacting with PIK3CB were extracted to construct the global regulatory network for functional annotation, illustrating the value of integrating epigenetic data for understanding complicated mechanisms. The cross-talking pathways of PIK3CB revealed that molecular functions of downregulated PIK3CB in AD were derived from apoptosis, axon guidance, and FoxO signaling pathway. The vulnerability of such pathways appears to be strikingly apparent at low PIK3CB levels, subsequently contributing to the onset of AD in a variety of pathogenic mechanisms [62, 72, 77, 88]. According to the AUC of 71.7%, low PIK3CB exhibited a good diagnostic performance in AD prediction, indicating that PIK3CB may be capable of a genetic risk factor of AD. Consistently, recent evidence from adult drosophila melanogaster suggests that human A*β* peptide is a candidate site for PI3K phosphorylation, the toxicity of which can be suppressed by co-expression of PI3K [11]. Future *in vivo* or *in vitro* experiments are needed to verify the mechanistic pathways of low PIK3CB-mediated AD neurodegeneration that are proposed in this in silico research.

## 5. Conclusion

In aggregate, integrative genomic analysis is an effective approach to uncover pleiotropic roles of PIK3CB underlying AD development. Our findings lend strong support to the notion that low PIK3CB expression is involved in the pathogenesis of AD through apoptosis, axon guidance, and FoxO signaling pathway.

## Data Availability

The data supporting the findings of this work are publicly available in GEO database (GSE109887, https://www.ncbi.nlm.nih.gov/gds/?term=GSE109887) and can also be obtainable from the authors upon reasonable request.
